# Lactobacilli Interfere with *Streptococcus pyogenes* Hemolytic Activity and Adherence to Host Epithelial Cells

**DOI:** 10.3389/fmicb.2016.01176

**Published:** 2016-07-29

**Authors:** Sunil D. Saroj, Lisa Maudsdotter, Raquel Tavares, Ann-Beth Jonsson

**Affiliations:** Department of Molecular Biosciences, The Wenner-Gren Institute, Stockholm UniversityStockholm, Sweden

**Keywords:** *Streptococcus pyogenes*, *Lactobacillus*, streptolysin, microbiota, adherence

## Abstract

*Streptococcus pyogenes* [Group A streptococcus (GAS)], a frequent colonizer of the respiratory tract mucosal surface, causes a variety of human diseases, ranging from pharyngitis to the life-threatening streptococcal toxic shock-like syndrome. Lactobacilli have been demonstrated to colonize the respiratory tract. In this study, we investigated the interference of lactobacilli with the virulence phenotypes of GAS. The *Lactobacillus* strains *L. rhamnosus* Kx151A1 and *L. reuteri* PTA-5289, but not *L. salivarius* LMG9477, inhibited the hemolytic activity of *S. pyogenes* S165. The inhibition of hemolytic activity was attributed to a decrease in the production of streptolysin S (SLS). Conditioned medium (CM) from the growth of *L. rhamnosus* Kx151A1 and *L. reuteri* PTA-5289 was sufficient to down-regulate the expression of the *sag* operon, encoding SLS. The *Lactobacillus* strains *L. rhamnosus* Kx151A1, *L. reuteri* PTA-5289, and *L. salivarius* LMG9477 inhibited the initial adherence of GAS to host epithelial cells. Intriguingly, competition with a combination of *Lactobacillus* species reduced GAS adherence to host cells most efficiently. The data suggest that an effector molecule released from certain *Lactobacillus* strains attenuates the production of SLS at the transcriptional level and that combinations of *Lactobacillus* strains may protect the pharyngeal mucosa more efficiently from the initial colonization of GAS. The effector molecules released from *Lactobacillus* strains affecting the virulence phenotypes of pathogens hold potential in the development of a new generation of therapeutics.

## Introduction

A range of bacterial communities including commensals and pathogens colonize the nasopharynx. The pharyngeal inhabitant *Streptococcus pyogenes* [group A streptococcus (GAS)], an adapted human pathogen, a common colonizer of the mucosa of the mouth, nose, and pharynx, is among the many pathogens that most often colonize their host asymptomatically and only occasionally cause disease. Local pharyngeal infection with GAS manifests as pharyngitis and, if spread from the local site, can cause the systemic diseases sepsis, streptococcal toxic shock syndrome, and necrotizing fasciitis (Luca-Harari et al., 2009). *S. pyogenes* produces a wide array of virulence factors, enabling it to adhere, invade, and spread within the human host (Cunningham, 2008). One of the characteristic features of *S. pyogenes* is the ability to lyse red blood cells (RBC), referred to as β-hemolysis

. *S. pyogenes* produces two different hemolysins/streptolysins, streptolysin S (SLS) and streptolysin O. The reason why *S. pyogenes* sometimes causes disease is not entirely understood, but both bacterial virulence factors and host factors are thought to contribute (Cole et al., 2011). The microbiota is one such host factor that needs further investigation. Attachment to epithelial cells is the crucial initial step of colonization because non-adherent GAS is removed by mucus and saliva flow. Additionally, bacterial interaction with the epithelial cells elicits multiple responses in the host cells, including cell signaling events, and modification of the host cell transcriptome (Nakagawa et al., 2004). The host responses regulate the bacterial colonization and play a significant role in the pathogenesis of the infection (Ribet and Cossart, 2015).

The microbiota prevents colonization with pathogenic bacteria and represents an important first line of defense. Mechanisms describing the probiotic effects of *Lactobacillus* strains include upregulation of mucin production in the host cells, interference with host pattern recognition receptors, competition for essential metabolites, production of antibacterial molecules, and co-aggregation between the bacteria of the microbiota and invading pathogenic bacteria, leading to interference with pathogen adherence to host cells (Lebeer et al., 2010; Reid et al., 2011; Lievin-Le Moal and Servin, 2014). *Lactobacillus* species are known to play a significant role in protection against many gastrointestinal and urogenital pathogens (Osset et al., 2001; Ostad et al., 2009). However, less is known about their antagonistic capacity against oral-pharyngeal pathogens. Additionally, the detailed mechanisms behind the anti-adhesive properties of lactobacilli and their effect on the expression of virulence-associated genes are far from fully understood.

Different species of *Lactobacillus* are part of the microbiota of the mucosal membranes in the pharyngeal tract (Badet and Thebaud, 2008). The study, therefore, aimed to investigate whether *Lactobacillus* strains interfere with the expression of the virulence-associated factors of *S. pyogenes* and its ability to adhere to pharyngeal epithelial cells.

## Materials and Methods

### Bacterial Strains and Growth Conditions

All *Lactobacillus* strains were isolated from healthy human individuals. *Lactobacillus rhamnosus* Kx151A1, isolated from a gastric biopsy, has been described previously (Roos et al., 2005). The *Lactobacillus* salivarius LMG9477, a type strain from the Belgian Coordinated Collections of Micro-organisms (BCCM), and *Lactobacillus reuteri* ATCC: PTA-5289, a kind gift from BioGaia AB, Stockholm were isolated from the oral cavity and have been described previously (de Klerk et al., 2016). Lactobacilli were grown on Rogosa agar plates and cultured in MRS broth (Oxoid, Thermo Fisher Scientific, Hampshire, UK). *S. pyogenes* S165 (GAS), serogroup *emm6*, was isolated from a septic patient (Sjolinder et al., 2008). GAS was grown on GC agar (Acumedia, Lansing, MI, USA) containing Kellogg’s supplement (Kellogg et al., 1963). All bacteria were cultured at 37°C and 5% CO_2_.

### Cell Lines and Culture Conditions

The pharyngeal epithelial cell lines FaDu (ATCC: HTB-43), and Detroit 562 (ATCC: CCL-138) were cultured in DMEM with GlutaMAX and pyruvate (Invitrogen, Carlsbad, CA, USA) supplemented with 10% heat-inactivated fetal bovine serum (Sigma–Aldrich, St. Louis, MO, USA). The cells were maintained at 37°C and 5% CO_2_ in a humidified environment. In all experiments, epithelial cells were seeded into cell culture plates the day before experiments to reach 90% confluence. The cell medium was replaced with serum-free DMEM half an hour before the experiments.

### Conditioned Media (CM)

Lactobacilli were grown on Rogosa agar plates. The bacteria were scraped off, washed twice with Todd–Hewitt broth (THB), and resuspended in THB. From this suspension, the growth of lactobacilli was initiated by adjusting to A_600_ ≈0.1 in THB. The growth was monitored until the density reached A_600_ ≈ 1.0. The lactobacilli were removed by centrifugation. The supernatant was passed through a 0.2 μm filter, adjusted to pH 7.8 and supplemented with 2% tryptone to obtain the Conditioned Media (CM, Saroj and Rather, 2013).

### Hemolytic Activity

Group A streptococcus was cultured in THB, co-incubated with lactobacilli, or in CM. For co-incubation, GAS and lactobacilli from agar plates were suspended in THB at A_600_ = 0.1 in the proportion of 1:1. At the desired time point, the bacteria were harvested by centrifugation; the supernatants were passed through a 0.2 μm filter and used for the hemolytic assay. RBCs for hemolytic assays were prepared from horse blood (Håtunalab AB, defibrinated) by washing three times in phosphate buffered saline (PBS) and diluting to 2% in PBS. The supernatants were diluted 1:10, mixed 1:1 with RBCs and incubated at 37°C in 5% CO_2_ for 1 h. The RBCs were removed by centrifugation at 2000 *g* for 3 min, and the supernatant was analyzed at A_404_ for hemolysis. Hemolytic activity was reported as the percentage relative to water alone. The SLS-mediated hemolytic activity was confirmed using trypan blue and cholesterol binding assays as SLS gets inhibited in the presence of trypan blue and SLO by cholesterol (Sierig et al., 2003). For all assays, the hemolytic activity was examined in the presence of trypan blue and cholesterol. The hemolytic activity of GAS in lactobacilli supernatants was measured by harvesting the supernatants at 1 h intervals until the growth reached the stationary phase. For hemolytic activity of GAS supernatant in co-incubation with CM, supernatant from the growth of GAS in THB from A_600_ ≈ 0.1 to A_600_ ≈ 1.0 was obtained. The supernatant was mixed with THB, or with CM from *L. salivarius* (L.sa), *L. rhamnosus* (L.rh), or *L. reuteri* (L.re) in a proportion of 1:1 and incubated for 30 min at 37°C with 5% CO_2_ prior to measuring the hemolytic activity.

### Viable Count

To asses the viability of GAS, viable count was performed by serial dilutions and plating on GC agar. In co-incubation experiments, the bacteria could be distinguished by colony size; GAS formed colonies larger than lactobacilli.

### Quantitative PCR Assays

Group A streptococcus was grown alone or co-incubated with lactobacilli in THB or in CM to A_600_ ≈ 1.0. The cells were harvested by centrifugation and total RNA was isolated using an RNeasy mini kit (Qiagen) after treatment with mutanolysin for 1 h. Total RNA 200 ng was reverse transcribed to cDNA using SuperScript VILO^TM^ master mix (ThermoFisher Scientific). The primers sagA-RT-(F) 5′GCTACTAGTGTAGCTGAAACAACTCAA-3′ and sagA-RT-(R) 5′AGCAACAAGTAGTACAGCAGCAA-3′ were used for the qPCR. The following conditions were used: an initial denaturation step at 95°C for 10 min; 45 cycles at 95°C for 15 s and 60°C for 1 min. The fold change was measured in relation to the housekeeping gene *gyrA* using the primers gyrA-RT-(F) 5′CGACTT GTCTGAACGCCAAAGT3′ and gyrA-RT-(R) 5′ATCACGTTCCAAACCAGTCAAAC3′.

### Luciferase Assay

The transcriptional fusion for the luciferase assay was prepared using pKSM720, which encodes a promoterless firefly luciferase (Almengor et al., 2007). The 400 bp region upstream of the *sag* operon was amplified using the primers sagAPro-F- 5′CCTGAGGATCCGTATTAGCGAGGTAAAG3′ and sagAPro-R- 5′AGTCACTCGAGAAGGTTTACCTCCTTATC3′. The fragments were gel purified, digested with BamHI and XhoI and cloned into the BglII and XhoI site of pKSM720. The resulting construct, pKSM720-sagApro-luc, was electroporated into GAS. Spectinomycin at 50 μg/ml was used to maintain the plasmid. The GAS with the construct was grown in THB or CM from lactobacilli from A_600_ ≈ 0.1 to A_600_ ≈ 1.0, and the luciferase activity measured according to the manufacturer’s instructions (Promega). The assay was conducted in the absence of spectinomycin. The maintenance of the plasmid construct was confirmed from the viable counts on THY plates with or without spectinomycin.

### Adherence Assays

Adherence assays were performed as previously described (Maudsdotter et al., 2011). FaDu (ATCC: HTB-43) or Detroit 562 (ATCC: CCL-138) pharyngeal epithelial cells were cultured in a 48-well plate and were inoculated with GAS at an MOI of 100 or together with lactobacilli at an MOI of 100 for 2 h. In competition assays, GAS and lactobacilli were added simultaneously. In exclusion assays, lactobacilli were pre-incubated with epithelial cells for 2 h followed by washing to remove unbound bacteria before the addition of GAS. In displacement assays, GAS was pre-incubated with the epithelial cells for 2 h followed by washing before addition of lactobacilli. When mixtures of lactobacilli were used, an MOI of 100 for each strain was added to the epithelial cells. After incubation, unbound bacteria were removed by washing in the medium. Adhered bacteria were quantified from viable counts obtained by lysing the epithelial cells for 5 min in 1% saponin and plating on GC plates. The adherence of GAS alone was normalized to 1.

### Statistical Analysis

All the experiments were performed in triplicate and repeated three times. Analysis of variance (ANOVA) and Tukey’s HSD (honestly significantly different) test (Statistica) was used to analyze differences between the groups for statistical significance. Statistical analysis of ratios or relative values was performed on log ratios. A *p*-value below 0.05 was considered statistically significant. Error bars represent standard deviation.

## Results

### Certain Lactobacilli Inhibit the Hemolytic Activity of *S. pyogenes*

**FIGURE 1:**
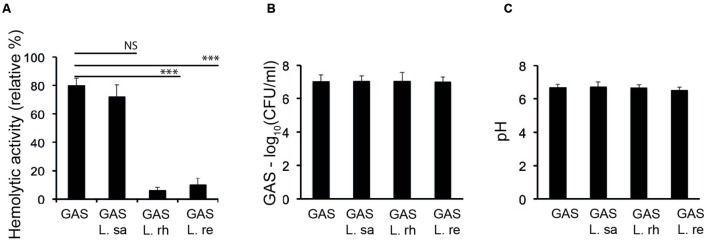
**Lactobacilli inhibit the hemolytic activity of Group A streptococcus (GAS).** **(A)** Hemolytic activity of GAS grown alone or co-incubated with *Lactobacillus rhamnosus* (L. rh), *L. reuteri* (L. re), or *L. salivarius* (L. sa) in THB. **(B)** Viable count of GAS **(C)** pH of the supernatant from the growth of GAS in THB alone or co-incubated with lactobacilli. Significant differences are marked with asterisks.

We investigated the influence of lactobacilli on the hemolytic activity of GAS. GAS was co-incubated with different *Lactobacillus* strains in THB and assayed for hemolytic activity. *L. rhamnosus* Kx151A1 (L. rh) and *L. reuteri* PTA-5289 (L. re) inhibited the hemolytic activity of GAS (**Figure 1A**). However, L. *salivarius* LMG9477 (L. sa) had no effect. The hemolytic activity was mediated by SLS because the presence of trypan blue, but not cholesterol, blocked the hemolytic activity (data not shown). To rule out the possibility that lactobacilli may inhibit the growth of GAS or affect the pH, we performed viable counts and measured the pH in the medium. The incubation of GAS with lactobacilli did not affect the survival of GAS (**Figure 1B**). Additionally, the pH of the growth media was not significantly affected during the co-incubation (**Figure 1C**). These results show that certain lactobacilli block the SLS-mediated hemolytic activity of GAS.

### *Lactobacillus* Growth Supernatant Inhibits the Hemolytic Activity of *S. pyogenes*

**FIGURE 2:**
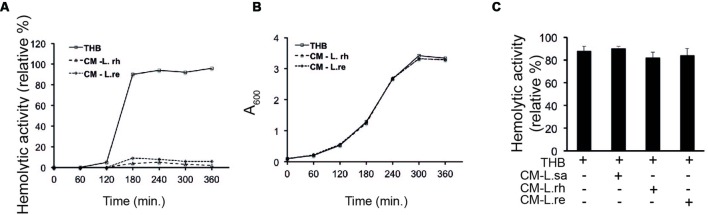
***Lactobacillus* growth supernatant inhibits the hemolytic activity of GAS **(A)** Hemolytic activity of GAS grown in THB, CM of *L. rhamnosus* (L. rh) and *L. reuteri* (L. re).** **(B)** Growth curve of GAS in THB and CM. **(C)** Hemolytic activity of GAS supernatant in co-incubation with CM. The supernatant obtained from the growth of GAS in THB was mixed with CM.

To examine whether the inhibition of hemolytic activity was due to molecules released from lactobacilli, a conditioned medium (CM) was prepared from the growth supernatants of lactobacilli. The hemolytic activity of GAS in the CM was assessed at regular intervals. The SLS-mediated hemolytic activity was inhibited in GAS grown in the CM from *L. rhamnosus* (L. rh) and *L. reuteri* (L. re) throughout the growth period (**Figure 2A**). As previously reported, hemolytic activity was detected during the logarithmic phase and reached a maximum in the stationary phase (Kinkel and McIver, 2008). The growth of GAS was not affected in the CM (**Figure 2B**). These results indicate that the inhibitory molecule is a component released from lactobacilli.

To examine whether the released effector molecule binds directly to SLS, supernatants from the growth of GAS in THB were mixed with the CM of lactobacilli and assayed for hemolytic activity. There was no observable difference in the hemolytic activity of the GAS supernatant mixed with fresh THB and the GAS supernatant mixed with CM from lactobacilli (**Figure 2C**). These data suggest that *Lactobacillus* strains release or secrete a soluble effector molecule that inhibits the hemolytic activity of GAS.

### Lactobacilli Interfere with the Expression of the *sag* Operon

**FIGURE 3:**
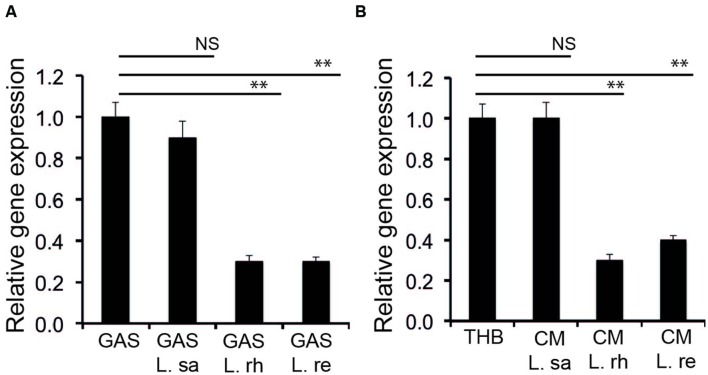
**Lactobacilli interfere with the expression of *sagA*.** **(A)** Expression of *sagA* during co-incubation of GAS with lactobacilli **(B)** Influence of the *Lactobacillus* effector molecule in CM on the expression of *sagA.* Significant differences are marked with asterisks.

Streptolysin S is chromosomally encoded by a contiguous nine-gene sag operon (Borgia et al., 1997; Molloy et al., 2011). The first gene of the operon is *sagA*, which encodes a 53 amino acid precursor. The expression of *sagA* during the co-incubation of GAS with lactobacilli was monitored by qPCR. There was no significant (*P* ≥ 0.05) change in the expression of *sag*A when GAS was co-incubated with *L. salivarius* (L. sa) (**Figure 3A**). However, when GAS was co-incubated with cultures of *L. rhamnosus* (L. rh) and *L. reuteri* (L. re), a significant (*P* < 0.01) decrease in *sag*A expression was observed. A similar effect was observed in the expression of *sag*A with GAS cultured in the CM of lactobacilli (**Figure 3B**). CM from *L. rhamnosus* (L. rh) and *L. reuteri* (L. re), but not *L. salivarius* (L. sa), inhibited *sagA* expression. The results indicate that the decrease in the hemolytic activity of GAS by lactobacilli is due to a decrease in the expression of the *sag* operon.

### Lactobacilli Interfere with the Promoter Activity of the *sag* Operon

**FIGURE 4:**
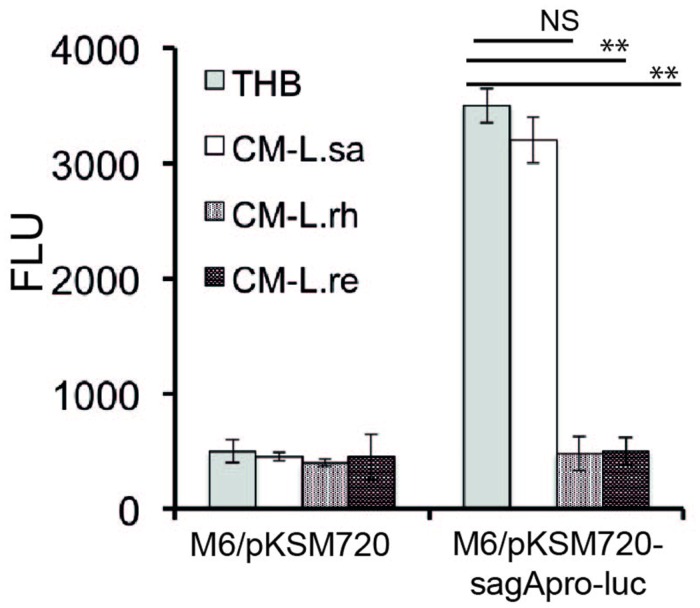
**Lactobacilli released component affects the promoter activity of *sagA*.** A luciferase assay was performed to determine the effect of the *Lactobacillus*-released effector molecule on the promoter activity of *sagA*. Significant differences are marked with asterisks.

To confirm that the effector molecule released from Lactobacillus inhibits the SLS at the transcriptional level, we constructed a transcriptional fusion of the promoter region of the *sag* operon to a promoterless luciferase reporter gene, *sag*Apro-luc. GAS harboring *sag*Apro-luc exhibited the expected luciferase activity when grown in THB. However, the luciferase activity was inhibited when the GAS with *sag*Apro-luc was cultured in the CM from *L. rhamnosus* (L. rh) and *L. reuteri* (L. re) (**Figure 4**), whereas the CM of *L. salivarius* (L. sa) had no effect. The data demonstrate that a lactobacilli-released effector molecule regulates the GAS SLS-mediated hemolytic activity at the transcriptional level.

### Lactobacilli Inhibit the Adherence of GAS to Host Epithelial Cells

**FIGURE 5:**
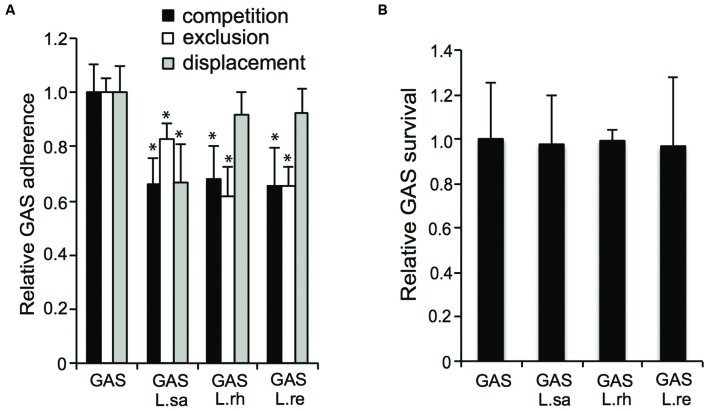
**Lactobacilli inhibit GAS adherence to pharyngeal epithelial host cells.** **(A)** Adherence of GAS to FaDu epithelial cells in a competition, exclusion and displacement assay **(B)** Recovery of GAS after co-infection with lactobacilli for 2 h. Significant differences are marked with an asterisk.

In addition to their effect on hemolytic activity, we also investigated if lactobacilli have an effect on the initial adherence of GAS to pharyngeal epithelial cells. All the *Lactobacillus* isolates, i.e., *L. rhamnosus* (L. rh), *L. reuteri* (L. re), and *L. salivarius* (L. sa), significantly (*P* ≤ 0.01) reduced the adherence of GAS by approximately 35% in a competition assay (**Figure 5A**). Additionally, all three *Lactobacillus* isolates significantly (*P* ≤ 0.01) inhibited streptococcal adherence when pre-incubated with the host cells for 2 h before the addition of GAS in exclusion assays. However, in the exclusion assays, a difference between the *Lactobacillus* isolates was observed: *L. rhamnosus* and *L. reuteri* inhibited streptococcal adherence by approximately 35%, whereas approximately 20% inhibition was observed with *L. salivarius*. Additionally, the ability of lactobacilli to displace already-adhered GAS was tested. *L. salivarius* significantly (*P* ≤ 0.01) inhibited streptococcal adherence by 35% in displacement assays. In contrast, neither *L. rhamnosus* nor *L. reuteri* displaced adhered GAS. In a previous study, we showed that co-incubating GAS with lactobacilli for longer time points (4–16 h) reduced GAS viability and thereby reduced the number of viable GAS adhered to host cells (Maudsdotter et al., 2011). However, co-incubating host cells with GAS and lactobacilli for 2 h did not affect streptococcal viability (**Figure 5B**). These data show that lactobacilli significantly reduce GAS adherence to epithelial cells.

### Effect of Different Combinations of *Lactobacillus* Strains on the Adherence of GAS

**FIGURE 6:**
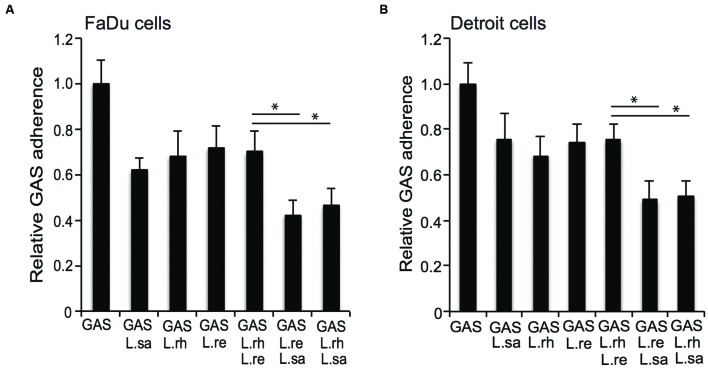
**Group A streptococcus adherence to epithelial cells in competition with different combinations of lactobacilli.** GAS adherence to **(A)** FaDu cells and **(B)** Detroit 562 cells after infection alone or together with *L. salivarius* (L.sa), *L. rhamnosus* (L.rh), and *L. reuteri* (L.re) for 2 h. Significant differences are marked with an asterisk.

Different combinations of *Lactobacillus* isolates were co-incubated with GAS to evaluate whether a combination of lactobacilli enhanced the anti-adhesive effect. A combination of *L. rhamnosus* (L. rh) or *L. reuteri* (L. re) with *L. salivarius* (L. sa) conferred a significantly (*P* ≤ 0.01) higher level of inhibition compared to co-incubation with any of the *Lactobacillus* isolates alone (**Figure 6A**). However, a combination of *L. rhamnosus* (L. rh) and *L. reuteri* (L. re) did not enhance the anti-adhesive effect compared to each species alone (**Figure 6A**). The effect of different combinations of lactobacilli on GAS adherence was tested in an additional cell line, pharyngeal epithelial Detroit 562 cells, and the same effects were observed (**Figure 6B**). Thus, a combination of *Lactobacillus* strains can increase the anti-adhesive effect.

## Discussion

Group A streptococcus and lactobacilli are both part of the pharyngeal microbiota. This study aimed to investigate how lactobacilli affect the virulence phenotypes of GAS in terms of hemolytic activity and adherence to host epithelial cells.

Lactobacilli are a heterogeneous group of bacteria. Additionally, it has been previously reported that different lactobacillus isolates vary widely in their ability to interfere with the virulence mechanisms of pathogens (Osset et al., 2001; Ekmekci et al., 2009; Ostad et al., 2009). GAS infections have been attributed to the variety of secreted and surface-bound virulence factors. One of the most important virulence factors encoded by GAS is SLS (Ginsburg, 1999). In addition to the ability to lyse RBCs, SLS has been shown to damage the cell membranes of lymphocytes, neutrophils, platelets, lysosomes, and mitochondria, indicating an important role for SLS in *S. pyogenes* pathogenesis (Ginsburg, 1999; Molloy et al., 2011). In this study, we found that lactobacilli were able to inhibit the hemolytic activity of GAS. Moreover, CM from the growth of lactobacilli was sufficient to inhibit SLS production. The transcriptional fusion and qPCR data revealed that this effect takes place at the transcriptional level. The secreted components from lactobacilli negatively regulate the promoter activity and down-regulate the *sag* operon.

Incoming pathogens must compete with the resident microbiota for nutrients and space to colonize the mucosal membranes in the body. Additionally, the microbiota modulate immune responses, produce inhibitory substances and prevent the attachment of pathogens and in this way protect the host from infections (Lebeer et al., 2010). Lactobacilli of different species belong to the microbiota of the pharynx, the gastric tract, and the urogenital tract and inhibit the adherence of many pathogens (Alexandre et al., 2014; Ribet and Cossart, 2015). In a few studies, the *Lactobacillus* components mediating adherence inhibition have been identified; lactobacilli produce biosurfactants (Servin, 2004; Rizzo et al., 2013), S-layer proteins (Velraeds et al., 1996, 1998; Spurbeck and Arvidson, 2010), and surface-located enolase (Ren et al., 2012), which have been reported to inhibit the adherence of different pathogens. However, the mechanisms by which *Lactobacillus* species inhibit the adherence of pathogens are still largely unknown. Some studies have reported a similar level of adherence inhibition when lactobacilli are added to host cells together with the pathogen as that observed when lactobacilli pre-colonize the host cells (Lee et al., 2003; Zhang et al., 2010). Other studies have reported a stronger inhibition of lactobacilli pre-colonizing the host cells when compared to lactobacilli added simultaneously with the pathogen (Vielfort et al., 2008). In this study, we demonstrate that *L. rhamnosus* Kx151A1, *L. reuteri* PTA-5289, and *L. salivarius* LMG9477 reduced the adherence of GAS to the host epithelial cells. In addition, we observed that *L. salivarius* conferred stronger protection when added in competition with GAS compared to when it pre-colonized the host cells. Further, *L. salivarius* was the only *Lactobacillus* species with the ability to displace already-adhered GAS. Our results highlight the differences between *Lactobacillus* species in the mechanism by which they confer colonization resistance as well as the effects they exert on host cells.

Various *Lactobacillus* species have previously been found to exhibit bactericidal activities against GAS in co-infection studies. In a previous study, we showed that co-incubation with lactobacilli for longer time points caused elevated lactic acid concentrations that are bactericidal against GAS and thereby reduce the number of viable GAS adhered to host cells (Almengor et al., 2007). In this study, we investigated the influence of lactobacilli on GAS adherence at a shorter time point at which GAS was not killed and observed inhibition of the initial adherence that was independent of compromised viability.

Thus, colonization with *Lactobacillus* species impairs GAS pathogenicity at different stages of infection, both by reducing the adherence at earlier stages and by affecting the viability after prolonged incubation and attenuating the hemolytic activity.

Probiotics are live microorganisms that confer health benefits on the host. *Lactobacillus* is a well-studied genus for probiotic use. Many criteria have been suggested for the selection of probiotics, including their ability to prevent the adherence of pathogens to target cells (Lee et al., 2003; Lebeer et al., 2010). The effects of probiotics are strain-specific, and the mechanisms of protection are largely unknown. The current study reveals that lactobacilli interfere with the virulence phenotypes of GAS in terms of SLS production and adherence to the host epithelial cells. Furthermore, a combination of lactobacilli has a greater effect in reducing the adherence of GAS to host epithelial cells and thus has an enhanced protective effect.

The findings may have a potential application in the use of lactobacilli strains as a probiotic for oral health purposes to combat GAS infections. Also, the secreted component from the lactobacilli holds a therapeutic value to fight GAS infections.

The identification of the *Lactobacillus*-secreted product interfering with the transcription of the *sag* operon is under study. Greater understanding of the molecular mechanisms by which probiotics function will help in developing enhanced future therapeutics.

## Author Contributions

Conceived and designed the experiments: SS, LM, A-BJ. Performed experiments: SS, LM, RT. Analyzed the data and wrote manuscript: SS, LM, A-BJ.

## Conflict of Interest Statement

The authors declare that the research was conducted in the absence of any commercial or financial relationships that could be construed as a potential conflict of interest.

## References

[B1] Alexandre Y. Le Berre R. Barbier G. Le Blay G. (2014). Screening of *Lactobacillus* spp. for the prevention of *Pseudomonas aeruginosa* pulmonary infections. *BMC Microbiol.* 14:107 10.1186/1471-2180-14-107 PMC4040502 24766663

[B2] Almengor A. C. Kinkel T. L. Day S. J. McIver K. S. (2007). The catabolite control protein CcpA binds to Pmga and influences expression of the virulence regulator Mga in the Group A *Streptococcus*. *J. Bacteriol.* 189 8405–8416. 10.1128/JB.01038-07 17905980 PMC2168945

[B3] Badet C. Thebaud N. B. (2008). Ecology of lactobacilli in the oral cavity: a review of literature. *Open Microbiol. J.* 2 38–48. 10.2174/1874285800802010038 19088910 PMC2593047

[B4] Borgia S. M. Betschel S. Low D. E. de Azavedo J. C. (1997). Cloning of a chromosomal region responsible for streptolysin S production in *Streptococcus pyogenes*. *Adv. Exp. Med. Biol.* 418 733–736. 10.1007/978-1-4899-1825-3_172 9331756

[B5] Cole J. N. Barnett T. C. Nizet V. Walker M. J. (2011). Molecular insight into invasive group A streptococcal disease. *Nat. Rev. Microbiol.* 9 724–736. 10.1038/nrmicro2648 21921933

[B6] Cunningham M. W. (2008). Pathogenesis of group A streptococcal infections and their sequelae. *Adv. Exp. Med. Biol.* 609 29–42. 10.1007/978-0-387-73960-1_3 18193655

[B7] de Klerk N. Maudsdotter L. Gebreegziabher H. Saroj S. D. Eriksson B. Eriksson O. S. (2016). Lactobacilli reduce *Helicobacter pylori* attachment to host gastric epithelial cells by inhibiting adhesion gene expression. *Infect. Immun.* 84 1526–1535. 10.1128/IAI.00163-16 26930708 PMC4862695

[B8] Ekmekci H. Aslim B. Ozturk S. (2009). Characterization of vaginal lactobacilli coaggregation ability with *Escherichia coli*. *Microbiol. Immunol.* 53 59–65. 10.1111/j.1348-0421.2009.00115.x 19291088

[B9] Ginsburg I. (1999). Is streptolysin S of group A streptococci a virulence factor? *APMIS* 107 1051–1059. 10.1111/j.1699-0463.1999.tb01509.x 10660134

[B10] Kellogg D. S. Jr. Peacock W. L. Jr. Deacon W. E. Brown L. Pirkle D. I. (1963). *Neisseria gonorrhoeae.* I. virulence genetically linked to clonal variation. *J. Bacteriol.* 85 1274–1279. 14047217 10.1128/jb.85.6.1274-1279.1963 PMC278328

[B11] Kinkel T. L. McIver K. S. (2008). CcpA-mediated repression of streptolysin S expression and virulence in the group A *Streptococcus*. *Infect. Immun.* 76 3451–3463. 10.1128/IAI.00343-08 18490461 PMC2493232

[B12] Lebeer S. Vanderleyden J. De Keersmaecker S. C. (2010). Host interactions of probiotic bacterial surface molecules: comparison with commensals and pathogens. *Nat. Rev. Microbiol.* 8 171–184. 10.1038/nrmicro2297 20157338

[B13] Lee Y. K. Puong K. Y. Ouwehand A. C. Salminen S. (2003). Displacement of bacterial pathogens from mucus and Caco-2 cell surface by lactobacilli. *J. Med. Microbiol.* 52, 925–930. 10.1099/jmm.0.05009-0 12972590

[B14] Lievin-Le Moal V. Servin A. L. (2014). Anti-infective activities of *Lactobacillus* strains in the human intestinal microbiota: from probiotics to gastrointestinal anti-infectious biotherapeutic agents. *Clin. Microbiol. Rev.* 27 167–199. 10.1128/CMR.00080-13 24696432 PMC3993101

[B15] Luca-Harari B. Darenberg J. Neal S. Siljander T. Strakova L. Tanna A. (2009). Clinical and microbiological characteristics of severe *Streptococcus pyogenes* disease in Europe. *J. Clin. Microbiol.* 47 1155–1165. 10.1128/JCM.02155-08 19158266 PMC2668334

[B16] Maudsdotter L. Jonsson H. Roos S. Jonsson A. B. (2011). Lactobacilli reduce cell cytotoxicity caused by *Streptococcus pyogenes* by producing lactic acid that degrades the toxic component lipoteichoic acid. *Antimicrob. Agents Chemother.* 55 1622–1628. 10.1128/AAC.00770-10 21245448 PMC3067128

[B17] Molloy E. M. Cotter P. D. Hill C. Mitchell D. A. Ross R. P. (2011). Streptolysin S-like virulence factors: the continuing sagA. *Nat. Rev. Microbiol.* 9 670–681. 10.1038/nrmicro2624 21822292 PMC3928602

[B18] Nakagawa I. Nakata M. Kawabata S. Hamada S. (2004). Transcriptome analysis and gene expression profiles of early apoptosis-related genes in *Streptococcus pyogenes*-infected epithelial cells. *Cell. Microbiol.* 6 939–952. 10.1111/j.1462-5822.2004.00412.x 15339269

[B19] Osset J. Bartolome R. M. Garcia E. Andreu A. (2001). Assessment of the capacity of *Lactobacillus* to inhibit the growth of uropathogens and block their adhesion to vaginal epithelial cells. *J. Infect. Dis.* 183 485–491. 10.1086/318070 11133381

[B20] Ostad S. N. Salarian A. A. Ghahramani M. H. Fazeli M. R. Samadi N. Jamalifar H. (2009). Live and heat-inactivated lactobacilli from feces inhibit *Salmonella* Typhi and *Escherichia coli* adherence to Caco-2 cells. *Folia Microbiol. (Praha)* 54 157–160. 10.1007/s12223-009-0024-7 19418255

[B21] Reid G. Younes J. A. Van der Mei H. C. Gloor G. B. Knight R. Busscher H. J. (2011). Microbiota restoration: natural and supplemented recovery of human microbial communities. *Nat. Rev. Microbiol.* 9 27–38. 10.1038/nrmicro2473 21113182

[B22] Ren D. Li C. Qin Y. Yin R. Li X. Tian M. (2012). Inhibition of Staphylococcus aureus adherence to Caco-2 cells by lactobacilli and cell surface properties that influence attachment. *Anaerobe* 18 508–515. 10.1016/j.anaerobe.2012.08.001 22922030

[B23] Ribet D. Cossart P. (2015). How bacterial pathogens colonize their hosts and invade deeper tissues. *Microbes Infect.* 17 173–183. 10.1016/j.micinf.2015.01.004 25637951

[B24] Rizzo A. Losacco A. Carratelli C. R. Domenico M. D. Bevilacqua N. (2013). *Lactobacillus plantarum* reduces *Streptococcus pyogenes* virulence by modulating the IL-17, IL-23 and Toll-like receptor 2/4 expressions in human epithelial cells. *Int. Immunopharmacol.* 17 453–461. 10.1016/j.intimp.2013.07.005 23892030

[B25] Roos S. Engstrand L. Jonsson H. (2005). Lactobacillus gastricus sp. nov., *Lactobacillus antri* sp. nov., *Lactobacillus kalixensis* sp. nov. and *Lactobacillus ultunensis* sp. nov., isolated from human stomach mucosa. *Int. J. Syst. Evol. Microbiol.* 55, 77–82. 10.1099/ijs.0.63083-0 15653856

[B26] Saroj S. D. Rather P. N. (2013). Streptomycin inhibits quorum sensing in *Acinetobacter baumannii*. *Antimicrob. Agents Chemother.* 57 1926–1929. 10.1128/AAC.02161-12 23318804 PMC3623334

[B27] Servin A. L. (2004). Antagonistic activities of lactobacilli and bifidobacteria against microbial pathogens. *FEMS Microbiol. Rev.* 28 405–440. 10.1016/j.femsre.2004.01.003 15374659

[B28] Sierig G. Cywes C. Wessels M. R. Ashbaugh C. D. (2003). Cytotoxic effects of streptolysin O and streptolysin S enhance the virulence of poorly encapsulated group a streptococci. *Infect. Immun.* 71 446–455. 10.1128/IAI.71.1.446-455.2003 12496195 PMC143243

[B29] Sjolinder H. Lovkvist L. Plant L. Eriksson J. Aro H. Jones A. (2008). The ScpC protease of *Streptococcus pyogenes* affects the outcome of sepsis in a murine model. *Infect. Immun.* 76 3959–3966. 10.1128/IAI.00128-08 18573900 PMC2519448

[B30] Spurbeck R. R. Arvidson C. G. (2010). *Lactobacillus jensenii* surface associated proteins inhibit *Neisseria gonorrhoeae* adherence to epithelial cells. *Infect. Immun.* 78 3103–3111. 10.1128/IAI.01200-09 20385752 PMC2897381

[B31] Velraeds M. M. van de Belt-Gritter B. van der Mei H. C. Reid G. Busscher H. J. (1998). Interference in initial adhesion of uropathogenic bacteria and yeasts to silicone rubber by a *Lactobacillus acidophilus* biosurfactant. *J. Med. Microbiol.* 47 1081–1085. 10.1099/00222615-47-12-1081 9856644

[B32] Velraeds M. M. van der Mei H. C. Reid G. Busscher H. J. (1996). Inhibition of initial adhesion of uropathogenic *Enterococcus faecalis* by biosurfactants from *Lactobacillus* isolates. *Appl. Environ. Microbiol.* 62 1958–1963. 8787394 10.1128/aem.62.6.1958-1963.1996 PMC167974

[B33] Vielfort K. Sjolinder H. Roos S. Jonsson H. Aro H. (2008). Adherence of clinically isolated lactobacilli to human cervical cells in competition with *Neisseria gonorrhoeae*. *Microbes Infect.* 10 1325–1334. 10.1016/j.micinf.2008.07.032 18761100

[B34] Zhang Y. C. Zhang L. W. Tuo Y. F. Guo C. F. Yi H. X. Li J. Y. (2010). Inhibition of *Shigella sonnei* adherence to HT-29 cells by lactobacilli from Chinese fermented food and preliminary characterization of S-layer protein involvement. *Res. Microbiol.* 161 667–672. 10.1016/j.resmic.2010.06.005 20600857

